# Spatial Aggregation Net: Point Cloud Semantic Segmentation Based on Multi-Directional Convolution

**DOI:** 10.3390/s19194329

**Published:** 2019-10-07

**Authors:** Guorong Cai, Zuning Jiang, Zongyue Wang, Shangfeng Huang, Kai Chen, Xuyang Ge, Yundong Wu

**Affiliations:** 1 Computer Engineering College, Jimei University, Xiamen 361021, China; guorongcai.jmu@gmail.com (G.C.);; 2 Fujian Collaborative Innovation Center for Big Data Applications in Governments, Fuzhou 350003, China

**Keywords:** LiDAR point cloud, deep learning, semantic segmentation, spatial structure information

## Abstract

Semantic segmentation of 3D point clouds plays a vital role in autonomous driving, 3D maps, and smart cities, etc. Recent work such as PointSIFT shows that spatial structure information can improve the performance of semantic segmentation. Motivated by this phenomenon, we propose Spatial Aggregation Net (SAN) for point cloud semantic segmentation. SAN is based on multi-directional convolution scheme that utilizes the spatial structure information of point cloud. Firstly, Octant-Search is employed to capture the neighboring points around each sampled point. Secondly, we use multi-directional convolution to extract information from different directions of sampled points. Finally, max-pooling is used to aggregate information from different directions. The experimental results conducted on ScanNet database show that the proposed SAN has comparable results with state-of-the-art algorithms such as PointNet, PointNet++, and PointSIFT, etc. In particular, our method has better performance on flat, small objects, and the edge areas that connect objects. Moreover, our model has good trade-off in segmentation accuracy and time complexity.

## 1. Introduction

The goal of semantic segmentation in a 3D point cloud is to give a semantic label to each point. The segmentation results can then be applied to autonomous driving, scene navigation, virtual reality, augmented reality, etc. However, due to the sparseness and disorder relationship of point cloud, there are many challenges in 3D point clouds’ semantic segmentation. In the past decade, researchers [1,2] have attempted to transform point clouds into regular data forms. The purpose is to transform the point cloud into data that can be processed by the general deep learning model. However, the transformation process may result in severe geometric information loss. Recently, researchers [3,4,5] tried to construct deep neural networks that allow raw point clouds, namely the coordinates and the intensities, as the input to networks. It is interesting to note that they usually use max-pooling to solve the disordered problem of point cloud. Nevertheless, max-pooling also leads to the loss of geometry information. As a consequence, the performance of max-pooling based method will reach a bottleneck.

Fortunately, recent work [6] revealed that the strategy of feature fusion from a local area may improve the discrimination ability of a point cloud feature. Therefore, in order to solve the problem of geometry information loss caused by max-pooling, we propose Spatial Aggregation Net (SAN) for point clouds’ semantic segmentation. Specifically, we use multi-directional convolution to extract the spatial structure of point clouds from different directions. In order to speed up the algorithm, we choose Octant-Search to select the neighbor points of each point. To sum up, we made two major contributions. First, SAN finds *K* neighbor points around the sampling point by Octant-Search. Second, we use multi-directional convolution to aggregate the local feature of neighbor points of each sampled point. Then, max pooling is used to handle the problem of disorder.

## 2. Related Work

Since LiDAR point clouds are composed of sparse and disordered points, traditional convolutional neural networks [7,8,9] with regular inputs are not suitable for point feature extraction. In the past decade, researchers have focused on converting 3D point clouds into a regular format such as images [1,10] and voxels [2,11]. Recently, raw point clouds based algorithms such as PointNet [3], PointNet++ [4], PointCNN [5], and PointSIFT [6] are proposed. The purpose is to reduce the scale of geometric information loss. Considering the type of input data, point clouds’ segmentation approaches can be divided into three categories, respectively as multi-views, voxel and raw point clouds based algorithms [12,13]. We’ll give a brief introduction of the three categories in the following paragraphs.

The first category is multi-view based methods. In order to solve the problem of data regularization, point clouds can be projected into an image plane according to the depth or the intensity values. Then, the task of feature extraction from a 3D point cloud can be transformed into 2D image processing. The performance of this strategy often depends on how to generate multi-view images. The simplest idea is to generate *K* projection views from virtual *K* camera poses, typically represented by Multi-view Convolutional Neural Networks (MVCNN) [14,15]. After the input point clouds are projected onto multiple images, each view can be used separately for semantic segmentation. Finally, all the segmentation results are re-projected back into the 3D space for label fusion. Since 2018, projection based methods, such as Points2Pix [16], View Inter-Prediction Generative Adversarial Networks(VIPGAN) [17], Pointwise Rotation-Invariant Networks(RPIN) [18], and RotationNet [19], have received widespread attention. These methods have achieved promising classification and segmentation accuracy on data sets such as ModelNet and ScanNet. Thus far, multi-view projection is still a hot topic in point clouds based deep learning approaches. However, the projection representation of point clouds still has some limitations—one of which is how to solve the problem of local geometric loss during the 3D to 2D data compression. Fortunately, there has been a breakthrough for handling local geometric loss. For example, Che et al. [20] proposed a novel Normal Variation Analysis (Norvana) that employed a region growing to a group point cloud on a smooth surface to obtain the segmentation results. More recently, they organized the point cloud data into a scan pattern grid, allowing the algorithm to process unorganized data [21]. By exploiting the scan pattern grid, the local geometric loss is greatly reduced during the process of point cloud segmentation. Moreover, to preserve local geometric information, Barnea et al. [22] integrate the range and the color content by using multiple cues. Song et.al. [23] introduce a large-scale benchmark suite with 3D annotations and 3D evaluation metrics that enable organized data for projection.

The second category is voxel based methods. Voxels are typically small units of point sets in 3D space. According to voxelization, point clouds can be divided into regular 3D subspaces. On the basis of 3D space meshing, 3D convolution filters, which are similar to 2D convolution networks, can be designed to perform feature fusion. A straightforward way to implement this strategy is to use 0–1 discrete values to mark whether there exists any point in the voxel or not. A typical method such as 3D ShapeNet [11] uses a binary voxel for 3D filtering. However, from two-dimensional convolution to three-dimensional convolution, the computational complexity may greatly increase. Actually, the size of each voxel acts as a trade-off between accuracy and complexity in the performance of point cloud segmentation. In other words, if we want to achieve higher segmentation accuracy, the size of each voxel should be smaller. However, the smaller the voxel grid, the higher the computational complexity. Therefore, researchers have attempted to transform the structure of voxel convolutional neural networks, such as Li et al. [24] and Tatarchenko et al. [25]. In spite of this, in voxel based convolutional networks, the non-uniformity of point clouds leading to high computational complexity is still a challenging problem. Convolution operations on voxels are often difficult when avoiding large amounts of redundant computation. The future work of voxel based method may focus on optimizing the convolution strategy and on constructing new voxel structures.

The third category is Raw point clouds based methods. Recently, researchers have been paying more and more attention to deep learning architectures which take raw point clouds as input. In this scheme, the coordinates, the intensity and the color of point clouds are combined as the input vector of deep neural networks. The most challenging task of raw point based method is to achieve order invariant. The milestone work for this category is PointNet [3], which was proposed in 2017. PointNet uses learnable transformation to regularize the point cloud. Based on extracting the local geometric features of the multi-layer perception, the global pooling is used to achieve order invariant. Since max pooling layers are applied across all points in the point clouds, it is difficult to capture local geometry feature for each point. In order to solve this problem, PointNet++ [4] introduced a hierarchical structure to improve the distinguishability of local point features. Actually, the idea is motivated by traditional 2D Convolutional Neural Netwoks(CNNs), which constructs a pyramid structure of point clouds. However, in the max-pooling layers of PointNet++, only the strongest reaction in features across a local or global region is preserved. This scheme may lose useful geometry information for the segmentation task. PointCNN [5] establishes a feature extraction scheme that ranges from a local area to global point clouds by selecting hierarchical representative points. However, when the point cloud is unevenly distributed, the selection of neighbor points may gather in a narrow area. As a consequence, the range of receptive field after several convolutions is limited. To this end, PointSIFT [6] selects the neighbor points from fixed orientations of the representative points. Therefore, the representative points can fully extract the surrounding spatial structural features. One of the disadvantages of PointSIFT is that the time complexity is high. Recently, SplatNet [26] proposed by Su et al. uses sparse bilateral convolution to implement hierarchical and spatial-aware feature learning, as well as joint 2D–3D reasoning. Point2Sequence [27] uses an implicit scheme, which employed Long Short-Term Memory(LSTM) [28] to the global pooling component, in order to extract point cloud features. At present, the raw point clouds based deep learning method mostly adopts an end-to-end structure, which can simultaneously extract local and global features. The main challenge is how to construct an efficient feature transfer mechanism during point cloud sampling to reduce excessive information loss. More recently, Wu et. al. [29] proposed a novel point convolution (PointCov), which treats convolution kernels as nonlinear functions of the local coordinates that comprised of weight and density functions. The reformulation process allows PointCov to dramatically scale up the network and significantly improve the performance.

## 3. The Proposed Approach

### 3.1. Directional Spatial Aggregation Module

**Figure 1:**
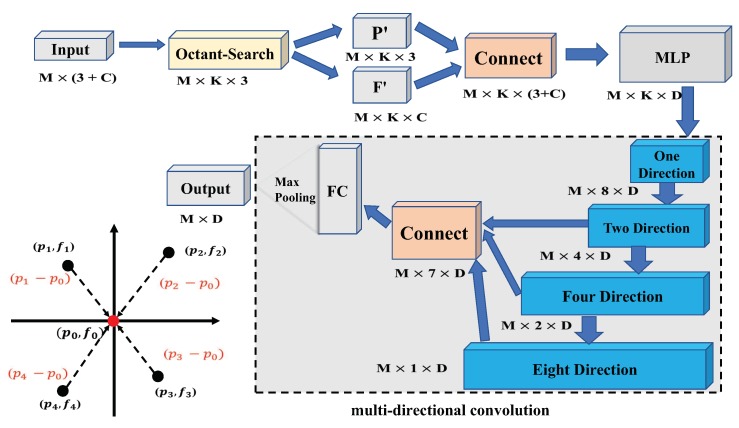
Illustration of the proposed directional spatial aggregation module.

Figure 1 shows the framework of the proposed Directional Spatial Aggregation (DSA), which takes points with coordinates (x,y,z) as the input of a deep neural network. Actually, our method is also motivated from the encode and decode scheme of U-net [30]. In particular, we use the Farthest Point Sampling (FPS) algorithm [31] to perform point down sampling. Let *C*, *D* be the number of feature maps for the input layer and the output layer, respectively. *M*, *K* are respectively the indices for the *M*th sampling points and the *K*th neighbor. Then, Octant-Search algorithm is employed to find *K* nearest neighbor points around the target point. The output is the 3D positions $P{}_{local}\in R{}^{M\times K\times 3}$ and the features $F{}_{local}\in R{}^{M\times K\times C}$ of selected points. Consequently, we can connect local coordinates with their features as the input vectors $F{}_{connect}\in R{}^{M\times K\times \left(3+C\right)}$. If the neighboring points don’t have feature vectors, the DSA module directly uses the local coordinates as the input feature $F{}_{connect}=P{}_{local}\in R{}^{M\times K\times 3}$. As for the new features of the *K* points, DSA uses four convolution operators to extract features from multiple directions. Finally, we use max-pooling to extract features in each direction. Then, $F{}_{out}\in R{}^{M\times D}$ is regarded as the output.

#### 3.1.1. Octant-Search for Neighbor Point Searching

**Figure 2:**
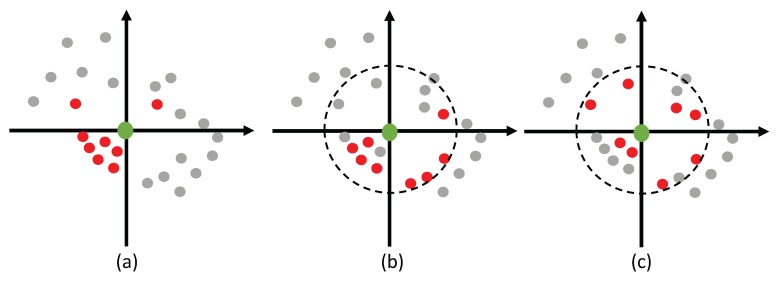
Illustration of the selection of neighbor points. (**a**) neighbor points selected by *K* nearest neighbor searching; (**b**) neighbor points selected by ball query searching; (**c**) neighbor points selected by octant-search.

In the point feature extraction process, neighbor points of each sampled point are expected to be more uniformly distributed in different directions. The purpose is that spatial information from different directions is helpful for point feature extraction. Actually, neighbor points selected by *K* nearest neighbor (KNN) searching algorithm may be concentrated in some local areas (as shown in Figure 2a). On the other hand, the ball query searching algorithm selects points randomly from a spherical area. As a consequence, the selected points are randomly distributed (as shown in Figure 2b). Neither of the two algorithms can select neighbor points uniformly, which cannot ensure robust features due to the loss of spatial information. To this end, we adopt octant-search scheme, which is different from the ball query searching and KNN searching. Given a selected point $p_{i}$, the neighbor 3D space of $p_{i}$ is partitioned into eight octants, which are centered at $p_{i}$. If there are some points that are far away from $p_{i}$, these points are regarded as useless points to represent $p_{i}$. For each octant, if there are not enough points within a given radius *r*, the represented point is replaced by $p_{i}$. Specifically, if the neighbor points of each octant are all replaced by $p_{i}$, it can be determined that there are no points in the neighbor area of $p_{i}$. On the other hand, for each octant, we select $\frac{K}{8}$ nearest points as the representative points of that octant (as shown in Figure 2c). It is worth noting that an octant-search algorithm selects points from multiple directions, which results in more uniformly distributed neighbor points.

#### 3.1.2. Multi-Directional Convolution

**Figure 3:**
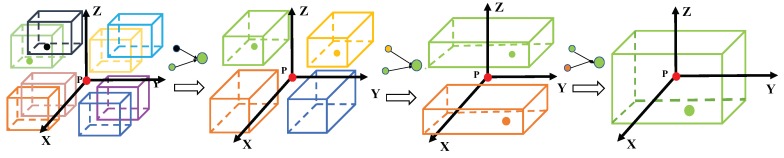
The details of multi-directional convolution.

In this section, we will analyze the motivation of the proposed multi-directional convolution. If max-pooling scheme is directly used for the point cloud, the information of a local geometric structure may lose because the pooling operation only retains maximum signals. As a consequence, it is very challenging for the network to perceive the local spatial structure of point clouds. However, in the task of point cloud semantic segmentation, local spatial structure always plays an important role to achieve promising results. To this end, we aim at retaining the local spatial structure of each sample point during the feature learning process. The purpose is to enhance the discriminant ability of point feature. As shown in Figure 3, the proposed multi-directional convolution is divided into four steps. First, we select *K* points around each sample point, where the *K* points are generated from an octant-search that is mentioned above. Note that there are $\frac{K}{8}$ points in each direction. For example, in our experiments, if we set *K* to be 32, then each direction has four points. The convolution operations then can be performed according to these neighbor points. In particular, the feature vectors of the four points in the same direction are fused to one vector via a convolutional operator. In the second step of our convolution scheme, the eight directions are fused to four directions, where points from *x*-axis directions are aggregated via a 2*1 convolutional operator. Similarly, the fusion strategy respectively passes though the *y*-axis and the *z*-axis. Through multi-directional convolution, we can get the convolution feature, which represents spatial structure information, of each point.

To perceive the local structure of point clouds, we perform four directional convolutions along different directions. As shown in Figure 3, four stages of directional convolution are respectively as:

(1) $$M{}_{1}=g\left[Conv{}_{1}\left(A{}_{1},M)]\in R{}^{8\times d}\right.\right.,$$

(2) $$M{}_{2}=g\left[Conv{}_{2}\left(A{}_{2},M{}_{1})]\in R{}^{4\times d}\right.\right.,$$

(3) $$M{}_{4}=g\left[Conv{}_{4}\left(A{}_{4},M{}_{2})]\in R{}^{2\times d}\right.\right.,$$

(4) $$M{}_{8}=g\left[Conv{}_{8}\left(A{}_{8},M{}_{4})]\in R{}^{d}\right.\right.,$$

where $A_{1}$, $A_{2}$, $A_{4}$, $A_{8}$ are convolution weights to be optimized. In this paper, we set g[∗] = ReLU(Batchnorm(∗)). After each convolution, the feature from local structure of several regions can be fused. For example, in Equation (2), we can get the local structure from four regions with a *y*-axis and *z*-axis.

After extracting the spatial structure information, we combined the features of the seven directions obtained by the last three convolutions to obtain a new matrix $M^{{}^{\prime }}$. Then, the information in these directions is fused by max-pooling via Equation (5), which is given as follows:

(5) $$M{}_{j}^{{}^{\prime \prime }}=\underset{i=1,...,7}{max}\left(M_{ij}^{{}^{\prime }}\right),j=1,...,d.$$

One can see that multi-directional convolution can extract information from all directions, in order to better extract the structural information around the sampling point. In addition, our network is simple and efficient. In fact, the section of experiments will show that the run time of the proposed method is faster than that of the state-of-the-art.

### 3.2. Overall Architecture

**Figure 4:**
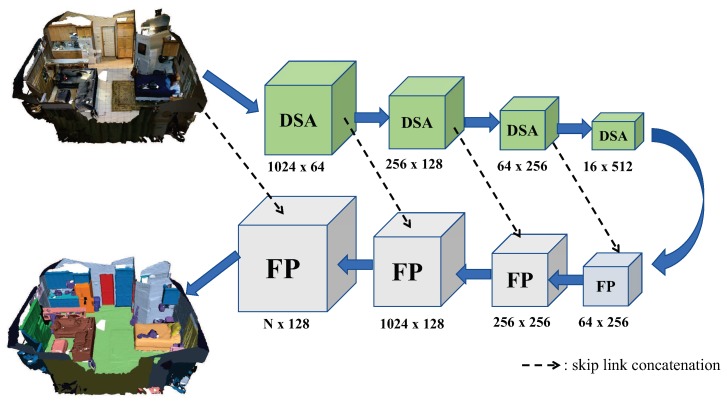
Illustration of the proposed end-to-end network architecture.

In order to fuse the features in the entire point set, we use a hierarchical structure that is able to combine small region features into semantic features that cover large spatial extent (as shown in Figure 4). The hierarchical structure is composed of several of the DSA modules and feature unencoding modules (FP), which are similar to PointNet++ [4]. The key layers in the structure are the DSA modules. The input of the first DSA is *N* points with three-dimensional coordinates, namely (*x*,*y*,*z*). The output of previous DSA is then regarded as the input of the next DSA. In Figure 4, the purpose of FP is to propagate features from selected points to each point of the raw point cloud. In particular, SAN adopts a hierarchical propagation strategy with distance based feature interpolation via level skip links, given in the dotted line in Figure 4. The network uses four FP modules to propagate the features to each point. As a result, the network generates the local features of *N* points and then uses a full connection layer to get the category of each point. In other words, the network can perform semantic segmentation of each point via the hierarchical structure.

## 4. Experiments

### 4.1. Experimental Setup and Implementation Details

As for the datasets, we used ScanNet and S3DIS to evaluate the performance of the proposed SAN. The experiments aim at comparing our SAN to the state-of-the-art, namely PointNet [3], PointNet++ [4], PointCNN [5], PointSIFT [6], and 3DCNN [32], in order to verify the efficiency of the proposed method. Thereinto, ScanNet is one of the most commonly used datasets in LiDAR point cloud segmentation. Specifically, ScanNet is a semantic scene labeling task that contains 1513 scanned scenes. In the experiments, we used 1201 scenes for training and 312 scenes for testing. The point clouds of all scenes are divided into fixed sizes to ensure the consistency of input. Basically, our approach follows the setting of PointNet++, which selects 8192 points for each sample. Moreover, we record the feed-forward time with the batch size of 5, then calculate the average run time for each method. In addition, the output of Equation (4) is compared with a DSA module to verify the performance of multi-direction fusion.

The second dataset is an S3DIS [33] indoor point cloud dataset, which consists of six regions with totaling 271 rooms. In our experiments, we used areas 1–5 for training, and then evaluated the performance in area 6. In all algorithms, the raw point clouds, namely the coordinate of each point, are regarded as input. All scene point clouds are divided into fixed size to ensure consistency of input point clouds. The input of networks followed the setting of PointNet, which selected 4096 points for each sample. In order to make a more precise comparison, we calculate the performance of SAN, PointNet++, PointSIFT on different categories in S3DIS datasets.

In order to make the results more intuitive, we selected typical experimental results to visualize the predictive models and the difference models. The training and the testing process for each method were conducted on NVIDIA 1080Ti with 12GB memory.

As for the hyper parameters of the proposed SAN, there are two key values that need to be determined, namely the number of DSA and the directions of each point for convolution. Firstly, it is worth noting that the DSA module is motivated from the SA module in PointNet++. Since PointNet++ recommended four SA modules for semantic segmentation, we chose four DSA modules in our architecture. Secondly, the number of directions is based on the experimental results achieved from a S3DIS dataset. Actually, we conducted three experiments where the number of directions have been set to be 4, 8 and 16, respectively. According to the experimental results, SAN with eight directions achieved the best performance.

### 4.2. The Results on ScanNet

The overall evaluation on ScanNet is given in Table 1. One can see the accuracy and the run time of all methods, where Ours1 means SAN without directional connection, and Ours2 denotes the complete SAN proposed in this paper. Note that, although Ours2 doesn’t achieve the best performance, it gives a good trade-off between accuracy and efficiency. Compared with PointNet++, the experimental results show that the proposed method achieves better performance, with almost as fast as Pointnet++. This phenomenon indicates that the scheme of multi-directional spatial aggregation does not significantly increase the time complexity. Since the proposed method is motivated by PointNet++, the results also reveal that max pooling plays an important role in aggregating features from different directions. Moreover, it proves that the component of multidirectional convolution can enhance the segmentation results. In other words, our method needs less run time to achieve the comparable accuracy with the state-of-the-art.

**Figure 5:**
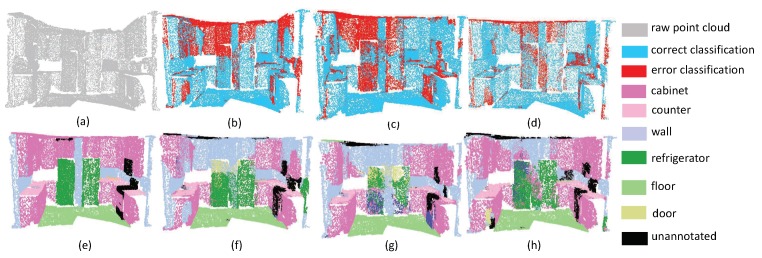
The plane segmentation results on Kitchen. (**a**) input, (**e**) ground truth, (**b**,**f**) the classification result by PointNet++, (**c**,**g**) the classification result by PointSIFT, and (**d**,**h**) the classification result by SAN.

**Figure 6:**
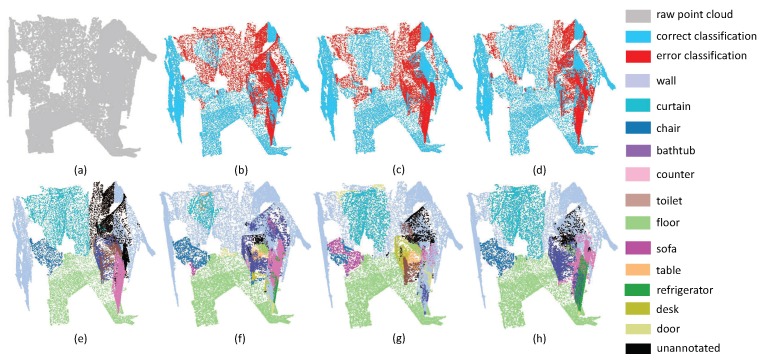
The plane segmentation results on Bedroom. (**a**) input, (**e**) ground truth, (**b**,**f**) the classification result by PointNet++, (**c**,**g**) the classification result by PointSIFT, and (**d**,**h**) the classification result by SAN.

In order to evaluate our algorithm more comprehensively, we also tried to test the performance on different kinds of scenes. The first experiment focuses on evaluating the accuracy of plane segmentation. It is noteworthy that each point on the plane has similar normal direction and magnitude. In this situation, the proposed local aggregate scheme can extract the *K* nearest points from different directions. In the pipeline of SAN, it is easy to extract plane features to construct a hierachical geometric structure. For example, as shown in Figure 5, there are many planes in this scene. Note that the segmentation of the cabinet is easily affected by wall areas. That being said, our algorithm achieved better performance than the PointNet++ and PointSIFT. One can see that in PointNet++ and pointSIFT, the border of planes is easily affected to nearby planes. In particular, the points of refrigerators are divided into doors and walls by PointNet++ and pointSIFT. This phenomenon reveals that the fusion of local convolution and spatial aggregation may enhance local geometry structure of point clouds. From Figure 6, we can draw the same conclusion since the proposed SAN can extract the curtains with the lowest false rate.

**Figure 7:**
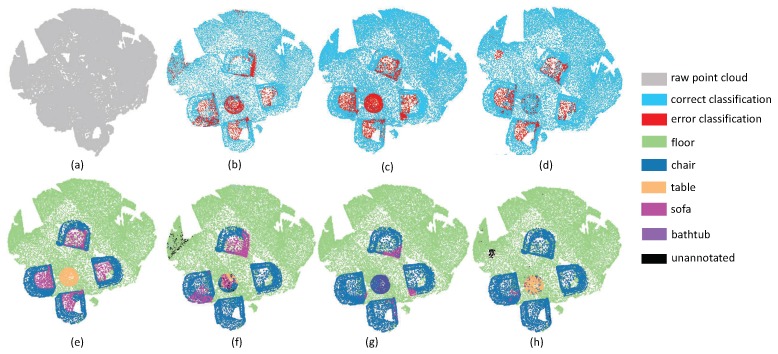
The small object segmentation results on Lounge. (**a**) input, (**e**) ground truth, (**b**,**f**) the classification result by PointNet++, (**c**,**g**) the classification result by PointSIFT, and (**d**,**h**) the classification result by SAN.

**Figure 8:**
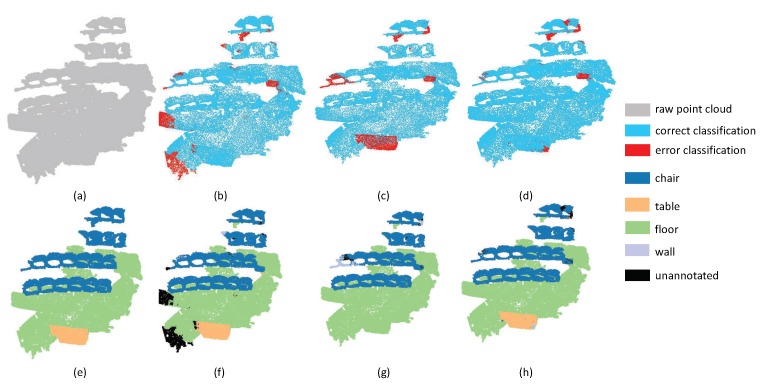
The small object segmentation results on Classroom. (**a**) input, (**e**) ground truth, (**b**,**f**) the classification result by PointNet++, (**c**,**g**) the classification result by PointSIFT, and (**d**,**h**) the classification result by SAN.

The second experiment focuses on evaluating the performance of small objects. Since small objects always have large inner-class variance, it is a challenging task to perform promising segmentation results. As shown in Figure 7, the scene includes four sofas around a table, where there are several pillows on each sofa. Since pillows and sofas are overlapping, it is very challenging to separate these objects. From the experimental results, one can easily see that all methods have promising results on the ground, since the plane areas are large and the boundaries are clear, while all methods including the proposed SAN fail to extract pillow areas precisely. It is worth noting that the SAN successfully extracted the table area, with only a small proportion of false labels. This phenomenon reveals that multi-directional convolution helps the aggregation of local information, which can further enhance the distinguishability of local features. Thus, the accuracy of small object segmentation results can be improved via the multi-directions scheme. From Figure 8, we can draw the same conclusion since the proposed SAN can extract the small table with the lowest false rate.

**Figure 9:**
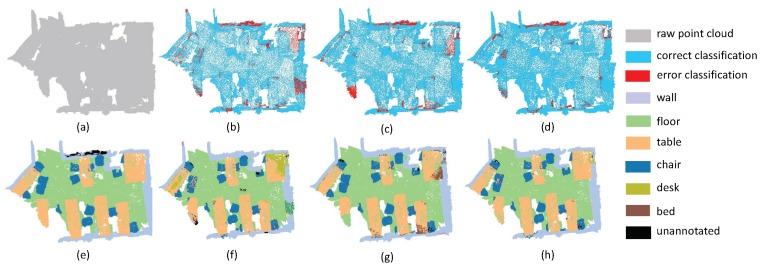
The edge segmentation results on Restaurant. (**a**) input, (**e**) ground truth, (**b**,**f**) the classification result by PointNet++, (**c**,**g**) the classification result by PointSIFT, and (**d**,**h**) the classification result by SAN.

**Figure 10:**
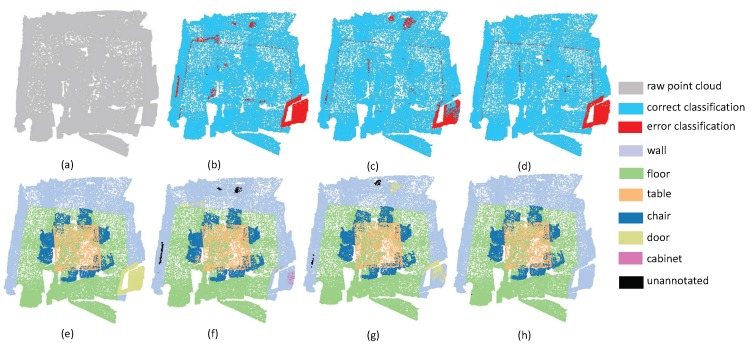
The edge segmentation results on ConferenceRoom. (**a**) input, (**e**) ground truth, (**b**,**f**) the classification result by PointNet++, (**c**,**g**) the classification result by PointSIFT, and (**d**,**h**) the classification result by SAN.

The third experiment aims at evaluating the robustness of edge area among different objects. Since the label information around irregular edge drastically changes, the segmentation results around edge areas are always unstable. As shown in Figure 9, there are several desks and chairs in a room. The task is challenging since there are some tight connection among tables, chairs and walls. From the experimental results, it can be seen that all the methods have promising results inside objects. However, PointNet++ and PointSIFT cannot achieve precise results on edge areas. Fortunately, our method has the lowest error rate of edge area segmentation. This phenomenon indicates that multi-directions convolution is helpful for the network to acquire the information of points in continuous edge, and then further improve the discrimination of edge area. From the segmentation results in Figure 10, we can see that SAN achieves better results than PointNet++ and PointSIFT at the edge of the table and the boundary between wall and floor. This phenomenon shows that SAN outperforms state-of-the-art methods on edge area among different objects.

**Figure 11:**
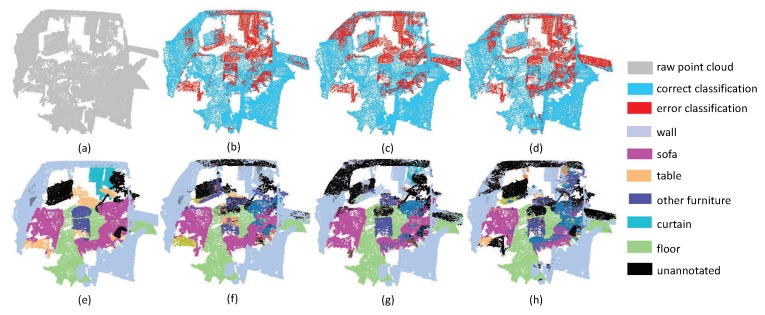
The complex scene segmentation results on LivingRoom1. (**a**) input, (**e**) ground truth, (**b**,**f**) the classification result by PointNet++, (**c**,**g**) the classification result by PointSIFT, and (**d**,**h**) the classification result by SAN.

**Figure 12:**
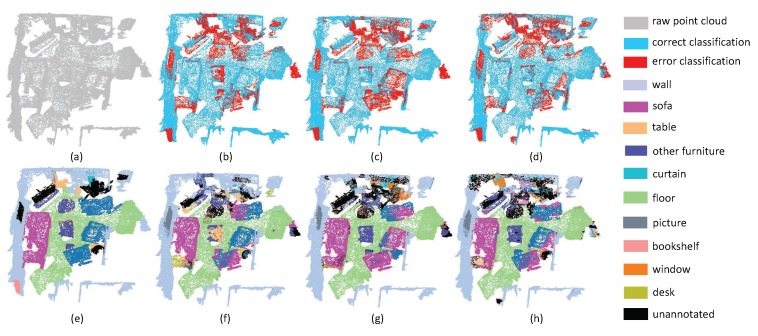
The complex scene segmentation results on LivingRoom2. (**a**) input, (**e**) ground truth, (**b**,**f**) the classification result by PointNet++, (**c**,**g**) the classification result by PointSIFT, and (**d**,**h**) the classification result by SAN.

The fourth experiment aims at evaluating the performance of complex scenes. We chose scenes that contain many objects in a small space. As shown in Figure 11, this room contains a TV, several sofas, several tables and some other furniture. It is challenging to achieve precise results in this situation. The results show that the performance of three methods, including the proposed SAN, are all unpromising. From Figure 12, we can see that the top layout of the scene is complex, since there are tables, chairs, furniture, curtains, walls in a small and crowded place. Moreover, most objects in this scene are placed closely. We can also find that SAN, PointNet++ and PointSIFT all fail in this scene. From the experimental results on ScanNet, we can draw a conclusion that SAN is an efficient method for LiDAR point cloud segmentation. However, the SAN network is still insufficient for complex scenes parsing.

### 4.3. The Results on S3DIS

Table 2 shows the overall results of PointNet, PointNet++, PointSIFT, and the proposed SAN on the S3DIS dataset, where SAN${}_{4}$, SAN${}_{8}$, and SAN${}_{16}$ represent the neighbor area of each sampled point that divided into four, eight and sixteen directions, respectively. The experimental results show that SAN${}_{8}$ achieves the best performance according to the accuracy. Namely, SAN with eight directions outperforms state-of-the-art methods including PointSIFT and PointNet++. The results also show that SAN with more directions doesn’t ensure better results, since SAN${}_{16}$ has worse performance than SAN${}_{8}$. The main reason may be that the space of 16 directions is over-separated, which results in unstable feature extraction. On the other hand, the results also show that convolution from four directions is un-sufficient to represent local geometry of sampled point, since the accuracy is even worse than SAN${}_{16}$.

Table 3 depicts the segmentation accuracy of each category. Note that SAN performs better than the other two algorithms in the categories of floors, ceilings, beams, columns, windows, tables, chairs, and sofas. In particular, SAN outperforms significantly PointNet++ and PointSIFT in the categories of beam and column. The reason may be that point features extracted by SAN acquires the spatial structure in the local area, especially in point clouds with plane and curved surface, such as beam and column.

**Figure 13:**
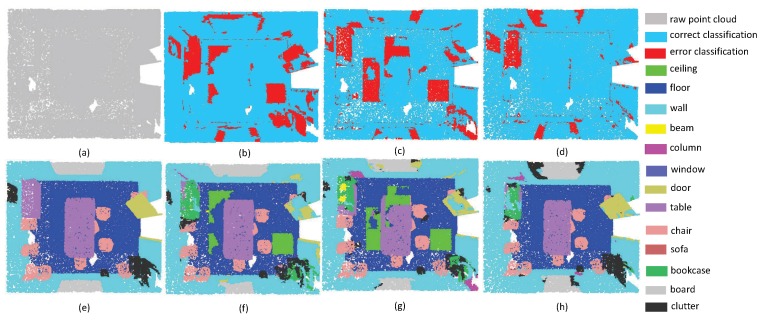
The segmentation results on ConferenceRoomS3D. (**a**) input, (**e**) ground truth, (**b**,**f**) the classification result by PointNet++, (**c**,**g**) the classification result by PointSIFT, and (**d**,**h**) the classification result by SAN.

**Figure 14:**
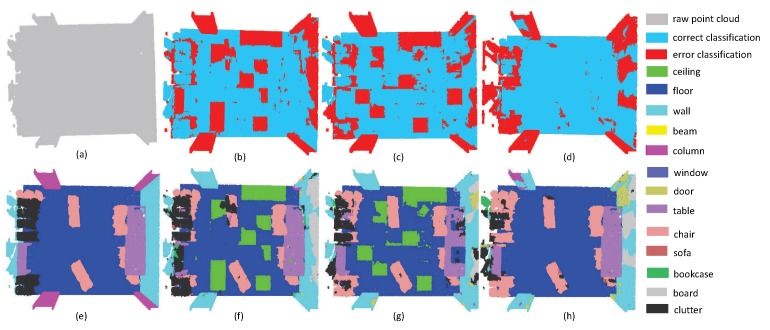
openspace on S3DIS. (**a**) input, (**e**) ground truth, (**b**,**f**) the classification result by PointNet++, (**c**,**g**) the classification result by PointSIFT, and (**d**,**h**) the classification result by SAN.

In order to make the segmentation results more intuitive, we chose typical examples, which are selected from different scenes, to visualize the point cloud parsing. The first experiment includes ConferenceRoom (Figure 13) and OpenSpace (Figure 14). These two point clouds are acquired from the office with tables and chairs inside the room. The results show that SAN achieved better performance in the categories of the table, chair and other planes. Although PointNet++ and PointSIFT are good at extracting large planes such as floor, they acquired larger proportion of wrong labels in table and chairs. However, in the boundary around these two rooms, SAN doesn’t achieve promising results. The main reason is that objects around the edge of the rooms are adjunct to the wall areas, resulting in the loss of generalized ability of point features.

**Figure 15:**
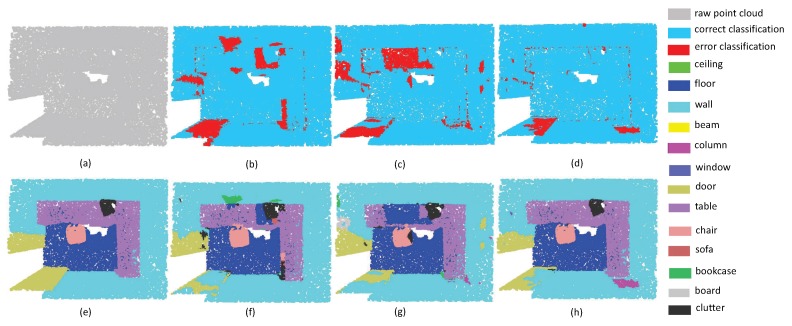
The segmentation results on OfficeS3D1. (**a**) input, (**e**) ground truth, (**b**,**f**) the classification result by PointNet++, (**c**,**g**) the classification result by PointSIFT, and (**d**,**h**) the classification result by SAN.

**Figure 16:**
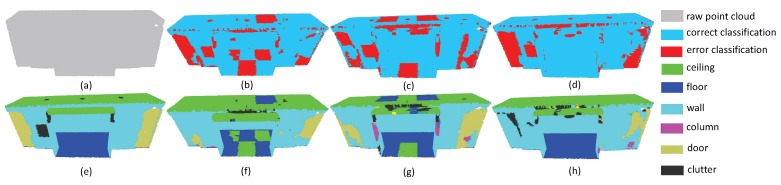
The segmentation results on HallWayS3D. (**a**) input, (**e**) ground truth, (**b**,**f**) the classification result by PointNet++, (**c**,**g**) the classification result by PointSIFT, and (**d**,**h**) the classification result by SAN.

In the second experiment, we choose a small space where objects are adjunct to each other. The results of Office and Hallway are respectively given in Figure 15 and Figure 16. One can see that SAN achieved the best results in these two scenes. In particular, our method is better than other methods in the category of table in Office (purple area in Figure 15) and floor (blue area in Figure 16). However, in the upper row of Figure 15d, one can see that SAN doesn’t perform good results in separate walls. The same thing happens in the wall area in Figure 16. This phenomenon shows that SAN may fail in adjunct objects with similar features.

**Figure 17:**
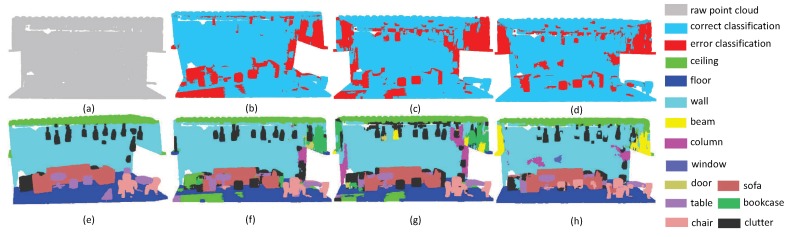
The segmentation results on LoungeS3D. (**a**) input, (**e**) ground truth, (**b**,**f**) the classification result by PointNet++, (**c**,**g**) the classification result by PointSIFT, and (**d**,**h**) the classification result by SAN.

In the third experiment, we try to evaluate the performance of SAN on a large space. As shown in Figure 17 (Lounge), there are many chandeliers’ sofas, tables and chairs in the room. In particular, these objects are randomly arranged. From the segmentation results, we can see that PointNet++, PointSIFT and SAN all fail in segmenting the droplight. As for tables and chairs, all three of the algorithms do not achieve promising results. Fortunately, SAN has better performance in the category of floor.

**Figure 18:**
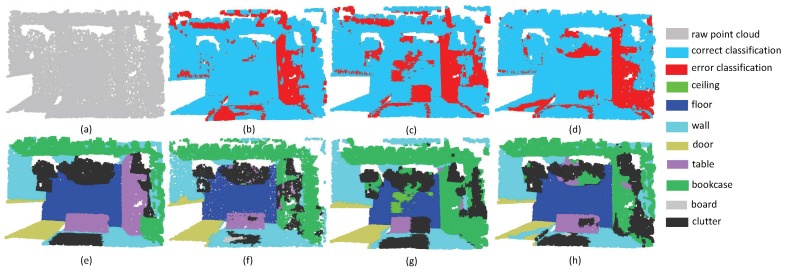
The segmentation results on CopyRoomS3D. (**a**) input, (**e**) ground truth, (**b**,**f**) the classification result by PointNet++, (**c**,**g**) the classification result by PointSIFT, and (**d**,**h**) the classification result by SAN.

**Figure 19:**
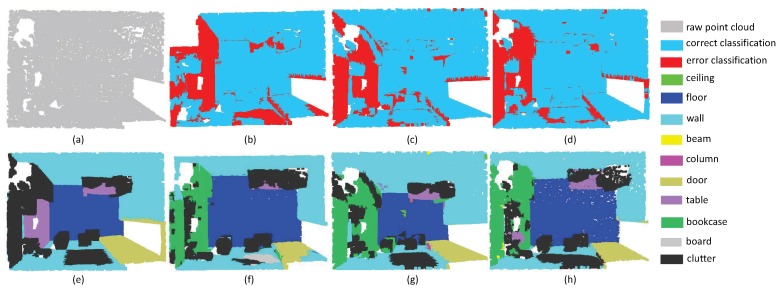
The segmentation results on PantryS3D. (**a**) input, (**e**) ground truth, (**b**,**f**) the classification result by PointNet++, (**c**,**g**) the classification result by PointSIFT, and (**d**,**h**) the classification result by SAN.

In the last experiment, we gave some examples that SAN is worse than the other algorithms. The results are shown in Figure 18 (CopyRoom) and Figure 19 (Pantry). In these two scenes, there are some appliances and furniture in the small room. From the accuracy, one can see that the proposed SAN has no obvious advantage over the other two methods. Actually, since most objects are placed near the walls, the feature extracted by multi-directions may be affected, which results in the loss of generality.

**Figure 20:**
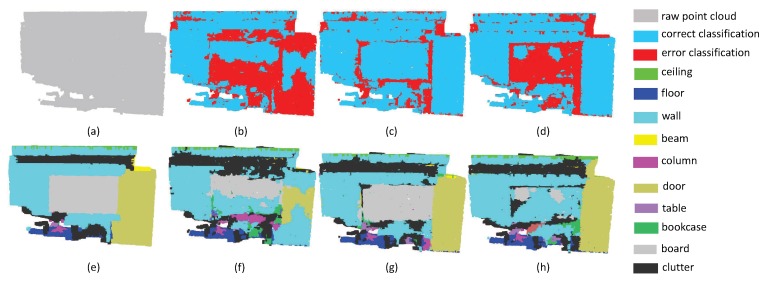
The segmentation results on OfficeS3D2. (**a**) input, (**e**) ground truth, (**b**,**f**) the classification result by PointNet++, (**c**,**g**) the classification result by PointSIFT, and (**d**,**h**) the classification result by SAN.

Although the proposed SAN achieved the highest accuracy on the dataset of S3DIS, in some cases, our method does not make sense. To analyze the main reason, we select typical fail examples to analyze the main reason. For example, in the category of board in Figure 20, the segmentation of our method is the worst, considering the accuracy of PointSIFT and PointNet++. It is worth noting that the board area is close to the wall, where the points from the board area are easily classified into wall with our method. Namely, given adjacent objects/areas with similar geometry structure, our feature fusion scheme based on multi-directions convolution may fail.

## 5. Conclusions

In this paper, we propose a novel Spatial Aggregation Net, which employed multi-directional convolution to aggregate the features of point cloud. The first contribution is that we use the spatial structure information of point cloud by eight neighborhoods from different directions. The second contribution is spatial structure information from multiple directions extracted by convolution; then, max pooling has been employed to achieve order invariance. The experimental results show that the proposed method has promising performances on small objects, plane objects, and the boundary between neighbor objects. That being said, SAN cannot directly be used in the task of large scale point cloud segmentation, considering the challenges of the training and the testing process. For example, the training time on NVIDIA 1080Ti of SAN is about 27 hours on ScanNet, which means that a large scale dataset may result in unacceptable training time. On the other hand, the input of SAN should be normalized/sampled to a specified number of points. As a consequence, large scale point cloud as input will result in severe information loss due to the process of sub-sampling. One potential solution is that a large scale point cloud can be divided into several subsets for distributed processing. Then, an efficient scheme to deal with a boundary effect should be designed. Our future work will focus on an efficient network that can deal with the segmentation task of more complex scenes.

## Figures and Tables

**Table 1:** Comparison of time and precision of different methods.

Methods	Accuracy (%)	Time (ms)
3DCNN [32]	70.0	-
PointNet [3]	73.9	7
PointNet++ [4]	84.5	52
PointCNN [5]	85.1	74
PointSIFT [6]	86.0	82
Ours1	84.9	52
Ours2	85.1	52

**Table 2:** The overall accuracy comparison of different methods on S3DIS.

Methods	Accuracy (%)
PointNet [3]	70.46
PointNet++ [4]	75.66
PointSIFT [6]	76.61
SAN${}_{4}$	74.16
SAN${}_{8}$	78.39
SAN${}_{16}$	76.31

**Table 3:** The comparison of different types of categories on S3DIS.

Category	SAN (%)	PointNet++ (%)	PointSIFT (%)
ceiling	98.83	92.46	92.84
floor	98.17	89.97	91.31
wall	83.43	86.92	87.31
beam	60.14	44.48	38.46
column	54.97	22.67	11.91
window	50.95	46.41	33.93
door	70.94	74.73	66.91
table	80.15	73.47	75.06
chair	86.09	84.61	84.76
sofa	72.42	68.56	63.04
bookcase	71.14	77.28	75.42
board	54.03	73.55	60.99
clutter	78.50	79.77	76.70
